# Aluminium alloyed iron-silicide/silicon solar cells: A simple approach for low cost environmental-friendly photovoltaic technology

**DOI:** 10.1038/srep17810

**Published:** 2015-12-03

**Authors:** Goutam Kumar Dalapati, Saeid Masudy-Panah, Avishek Kumar, Cheng Cheh Tan, Hui Ru Tan, Dongzhi Chi

**Affiliations:** 1 Institute of Materials Research and Engineering, A*STAR (Agency for Science, Technology and Research), 3 Research Link, Singapore 117602

## Abstract

This work demonstrates the fabrication of silicide/silicon based solar cell towards the development of low cost and environmental friendly photovoltaic technology. A heterostructure solar cells using metallic alpha phase (*α*-phase) aluminum alloyed iron silicide (FeSi(Al)) on *n*-type silicon is fabricated with an efficiency of 0.8%. The fabricated device has an open circuit voltage and fill-factor of 240 mV and 60%, respectively. Performance of the device was improved by about 7 fold to 5.1% through the interface engineering. The *α*-phase FeSi(Al)/silicon solar cell devices have promising photovoltaic characteristic with an open circuit voltage, short-circuit current and a fill factor (FF) of 425 mV, 18.5 mA/cm^2^, and 64%, respectively. The significant improvement of *α-*phase FeSi(Al)/*n*-Si solar cells is due to the formation p^+−^n homojunction through the formation of re-grown crystalline silicon layer (~5–10 nm) at the silicide/silicon interface. Thickness of the regrown silicon layer is crucial for the silicide/silicon based photovoltaic devices. Performance of the *α-*FeSi(Al)/*n*-Si solar cells significantly depends on the thickness of *α-*FeSi(Al) layer and process temperature during the device fabrication. This study will open up new opportunities for the Si based photovoltaic technology using a simple, sustainable, and los cost method.

Reliable, sustainable, and affordable renewable energy is critical to meet basic human needs^1^,^2^. Therefore, low cost solar cells are much sought-after for the next generation photovoltaic technology to meet large-scale electricity supply with low-carbon emission^1^. Globally there is an enormous effort to reduce the cost of the solar cells. Low cost solar cells can be realized either by increasing efficiency of the solar cells or through the reduction of cost per efficiency. Toward this, we have demonstrated iron-silicide/silicon based solar cells by using metallic α-phase aluminum alloyed iron silicide (FeSi(Al)) through sputter deposition technique for sustainable and environmental-friendly photovoltaic technology.

The ultimate goal of solar energy is to be economically competitive with fossil based electricity. Among different photovoltaic (PV) technologies, crystalline silicon based solar cells are the dominant one. Although the price of crystalline silicon solar cell and the modules has dropped significantly in the last few years, the cost per efficiency must go down even further^3^,^4^. To achieve parity with existing mains grid electricity prices, known as ‘grid parity’, lower material and process costs are also crucial^3^. The fabrication cost for solar cell modules includes the cost of the cell processing (20%), module processing (30%) and silicon substrate (50%)^4^. Therefore reducing the cost of Si wafer by minimizing the amount of crystalline Si can be considered as an effective method to reduce the overall cost of the solar cell modules^5^. On the other hand, developing novel structure and integration of advanced materials with silicon are key strategic approach to reduce cost per efficiency of the solar cells^3^,^4^,^5^,^6^,^7^,^8^. Silicon microwire and nanowire based solar cells^9^,^10^, heterojunction solar cells^11^,^12^, Schottky junction solar cells such as graphene/silicon^13^,^14^,^15^, CNT/silicon^16^, and 2D material/silicon based solar cells^17^ are few technologies that have demonstrated for the fabrication of high efficiency solar cell in a cost effective way.

The silicide materials are generally metallic in nature. Among several silicide materials, iron silicide appears to be a potential candidate for optical and electronic applications^18^,^19^,^20^,^21^,^22^. It exhibits metallic (*α*-phase) and semiconducting (*β*-phase) behavior based on their preparation conditions and structural property. The *β*-phase FeSi_2_ is the only one which is semiconducting in nature and there are several reports on *β*-phase FeSi_2_ and its promising optoelectronic properties^18^,^19^,^22^,^23^. Recently, semiconducting *β*-phase FeSi_2_ has been a material of keen interest for photovoltaic (PV) community owing to its numerous promising properties such as earth abundant, nontoxic, and suitable bang gap^19^,^24^,^25^,^26^,^27^,^28^,^29^. On the other hand, metallic *α*-phase FeSi_2_ has huge potential for practical application in circuits and devices^20^,^30^,^31^,^32^. The *α*-FeSi_2_ has electrical resistivity as low as 2 × 10^−4^ Ω-cm and can be used as electrode to form high quality ohmic contacts in *β*-FeSi_2_ based optoelectronic devices^20^,^30^,^31^. Moreover, *α*-phase FeSi_2_ has a high work function and is environmental stable. The metallic *α*-phase iron silicide has relatively high barrier height with *n*-type Si which makes it is a suitable candidate for a possible metal-semiconductor Schottky junction solar cells^33^.

There are few reports in literature where laser annealing has been used to fabricate *α*-FeSi_2_^20^,^31^. The *α*-phase FeSi_2_ has high transition temperature (937 °C and above), below the transition temperature, it shows *β*-phase FeSi_2_. This high transition temperature (>937  °C) possess several challenges towards its integration with other semiconductor material for device applications. In addition, the high transition temperature required to fabricate *α*-FeSi_2_ is expected to degrade interface quality which would be detrimental to device performance. Thus, it is desired to reduce the transition temperature of *α*- phase iron silicide. Fortunately, Al-alloyed iron silicide reduces the transition temperature significantly^26^,^34^. By controlling the Al composition and thickness of iron-silicide, it is possible to grow high quality *α*-phase FeSi(Al) alloy at 600 °C^35^. Furthermore, the thermal treatment of Al alloyed amorphous iron-silicide leads to the formation of uniformly regrown crystalline silicon layer at the silicide/silicon junction, which improved interface quality^35^. Even though, use of *α*-phase FeSi(Al) ternary alloy has huge potential for photovoltaic application, there has been no effort towards the integration of *α*-phase FeSi(Al) with silicon for photovoltaic devices.

Sputter deposition method provides high quality thin film over large area. Sputter grown thin film has a huge potential for solar energy harvesting application^22^,^24^,^26^,^27^,^28^,^29^,^35^,^36^,^37^. For large scale deployment of solar cells, a simple method with stable and reliable photovoltaic technology is required. In the present work, we demonstrate the fabrication of a novel solar cell using sputter grown α-phase FeSi(Al) ternary alloy and *n*-Si(100) substrate with a record efficiency (*Eff*) and fill factor (*FF*) of ~5.1% and 63.5%, respectively. It is worth to emphasize here that the fabricated solar cell does not have any antireflection coating or surface passivation. The proposed solar cell provides a possible solution towards the development of low cost environment friendly photovoltaic technology. The fabrication of this kind of solar cells is simple and requires few steps using widely accepted industrial tools and is capable of batch processing. At the same time, it also eliminates the use of expensive diffusion techniques and the toxic chemical process.

## Experiment

**Figure 1:**
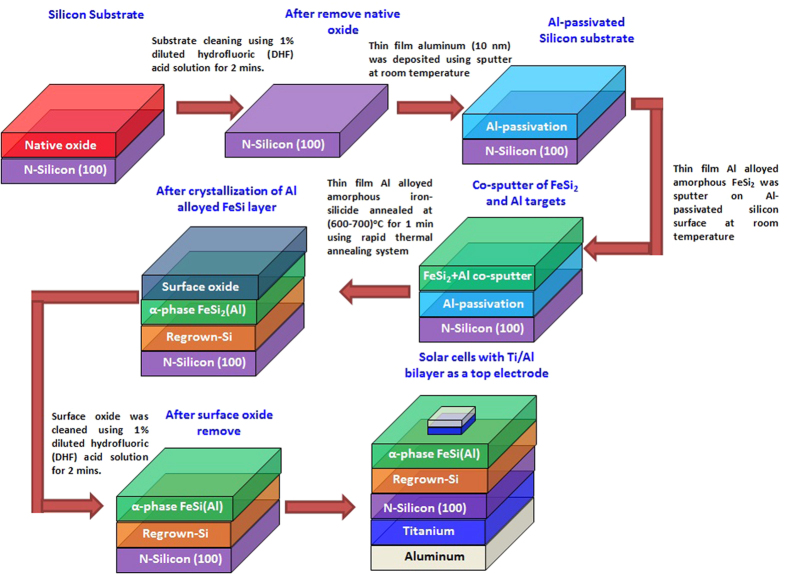
Schematic illustration of the steps involved in the fabrication of the *α*-FeSi(Al)/*n*-Si solar cell test structure.

A conventional magnetron-sputter and rapid thermal annealing system were employed to fabricate *α*-FeSi(Al)/*n*-silicon (*n*-Si) based solar cells. Firstly, Si substrates were dipped into diluted hydrofluoric acid solution (1%) for 3 mins to remove native oxide from the surface and then Si substrates were loaded immediately into a magnetron sputtering chamber. Prior to the amorphous iron silicide deposition, a thin aluminum (Al) interlayer (~10 nm) was deposited on *n-*Si substrates with a base pressure ~10^−7^ Torr to passivate Si surface. Subsequently, different thickness of Al alloyed iron silicide (FeSi_2_) was co-sputtered on Al-passivated *n*-Si substrate by using stoichiometry FeSi_2_ target at 100 W and Al target at ~2 W in an Ar ambient. Thickness of Al alloyed FeSi_2_ varied from 15 nm to 35 nm. The Al alloyed iron silicide coated Si samples were then subjected to rapid-thermal-annealing (RTA) in nitrogen ambient at temperatures of 600 °C and 700 °C for 60 s for the formation of *α*-FeSi(Al)/*n*-Si solar cells. After thermal treatment, native oxide from the surface of FeSi(Al) was removed by using diluted HF solution. High-resolution transmission electron microscopy (HRTEM) were employed to investigate the structural property of *α*-FeSi(Al)/*n*-Si structure. Energy dispersive X-ray (EDX) analysis was used to analyze composition of the Al alloyed iron silicide ternary layer. For the photovoltaic study, thin film Ti/Al double layer metal contact was sputtered to form an Ohmic front and back contact of *α*-FeSi(Al)/*n*-Si(100) solar cell. Figure 1 shows the schematic diagram of the steps involved in the fabrication of the *α*-FeSi(Al)/*n*-Si solar cell structure. Photovoltaic characteristics of the *α*-FeSi(Al)/*n*-Si solar cells were then investigated at AM 1.5 illumination using solar simulator. The external quantum efficiency (EQE) were measured by PVE300 (Bentham) IPCE Instrument equipped with a xenon/quartz halogen light source.

**Figure 2:**
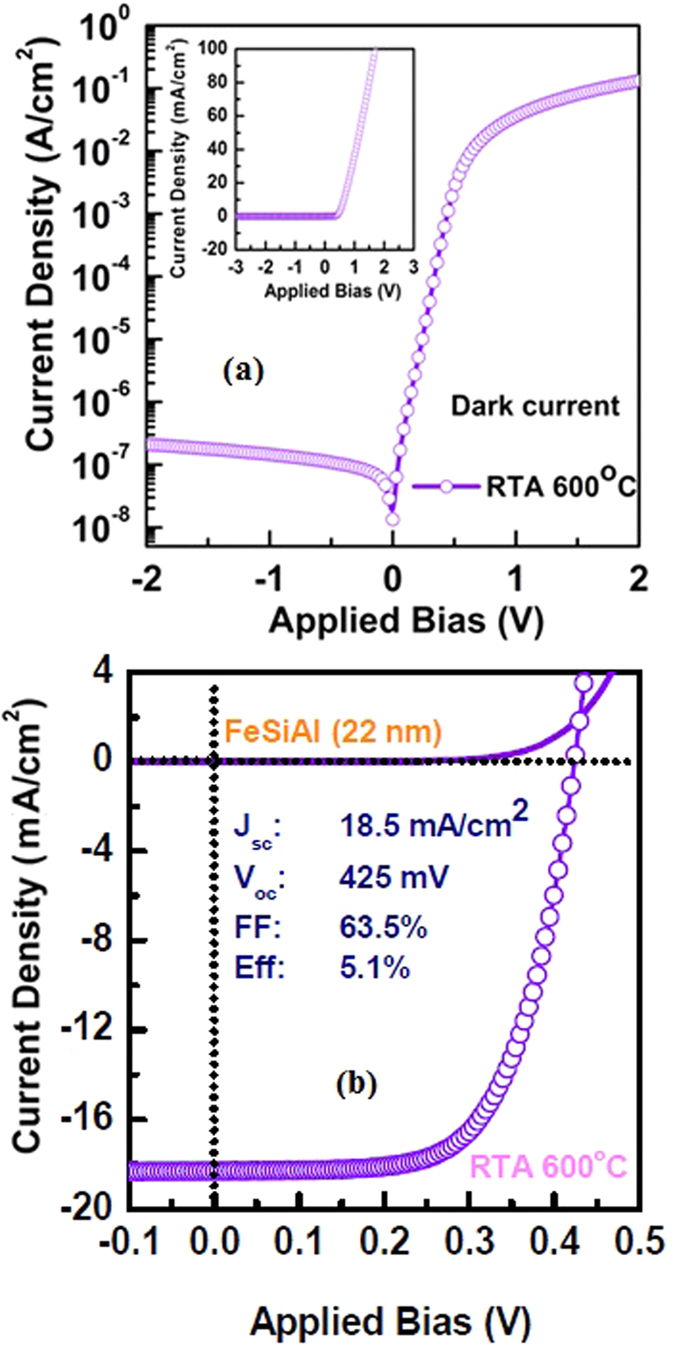
(**a**) Dark current-voltage characteristics of *α*-phase FeSi(Al)/*p*^+^-Si/*n*-Si solar cells. The current voltage characteristic shows typical *p*-*n* junction behavior with on/off current ratio of 10^6^. (**b**) Photovoltaic characteristics of *α*-phase FeSi(Al)/*p*^+^-Si/*n*-Si solar cells. The *α*-phase FeSi(Al) layer was formed after thermal treatment at 600 °C.

The performance of these solar cells was evaluated using current-voltage (I-V) and the EQE measurements. Figure 2(a) shows the dark current-voltage characteristics of the 22 nm thick *α*-FeSi(Al)/*n*-Si(100) solar cells fabricated after thermal treatment of Al-alloyed iron-silicide at 600 °C. A typical rectifying characteristic with the on/off rectification ratio of 10^6^ is observed confirming the formation of high quality diode. Figure 2(b) shows the current density-voltage (J-V) characteristics of 22 nm thick *α*-FeSi(Al)/*n*-Si(100) solar cell. A record open circuit voltage (*V*_*oc*_), short-circuit current (*J*_*sc*_), and a fill factor (FF) of 425 mV, 18.5 mA/cm^2^, and ~63.5%, respectively are observed for the device. Accordingly, this solar cell exhibits power conversion efficiency (PCE) of 5.1%, which is highest reported efficiency among all iron-silicide/silicon based solar cells. Furthermore, efficiency of the prepared solar cell at this paper is comparable to other reported results using a similar kind of device structure where they have used monolayer layer MoS_2_^17^ and graphene^11^,^13^,^14^,^15^ to form a Schottky junction solar cell with Si. It is worth to emphasize that the solar cell reported here uses only about 22 nm of *α*-FeSi(Al) to form the junction and utilizes simple steps using a simple conventional sputter deposition and rapid thermal annealing techniques for its fabrication.

**Figure 3:**
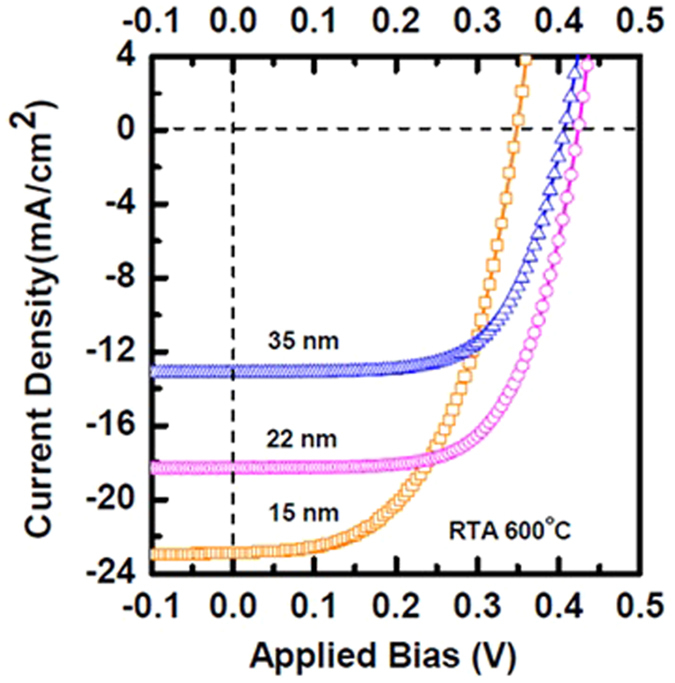
Photovoltaic characteristics of *α*-phase FeSi(Al)/*p*^+^-Si/*n*-Si solar cells with different thickness of *α*-phase FeSi(Al) layer. The *α*-phase FeSi(Al) layer was formed after thermal treatment at 600 °C.

To understand the impact of the thickness variation of *α*-FeSi(Al) layer on the performance of the solar cell in details, we fabricated solar cells with different *α*-FeSi(Al) layer thickness. Figure 3 shows the current density-voltage characteristics of the *α*-phase FeSi(Al)/*n*-Si solar cells with three different thickness of *α*-FeSi(Al) layer. The photovoltaic parameters of *α*-phase FeSi(Al)/*n*-Si solar cells with different thickness of *α*-phase FeSi(Al) layer were extracted using numerical method and tabulated in Table 1. It can be clearly observed that the *V*_*oc*_ and *J*_*sc*_ of the device significantly depend on the thickness of *α*-FeSi(Al) layer. With the decrease of *α*-FeSi(Al) layer thickness, current increases. This could be due to the increase of light intensity into the junction. However, as the thickness of *α*-FeSi(Al) layer decreased to 15 nm, the performance of the device drops significantly mainly due to the reduction of open-circuit voltage. This suggests the degradation of interface quality between silicide and silicon layer.

**Figure 4:**
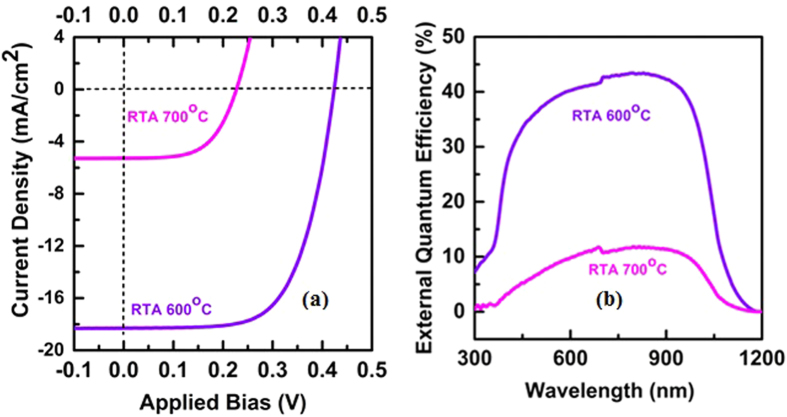
(**a**) Comparison of photovoltaic characteristics of *α*-phase FeSi(Al)/*p*^+^-Si/*n*-Si solar cells, where *α*-phase FeSi(Al) layer was formed after thermal treatment at 600 °C and 700 °C. The performance of the solar cells degraded significantly after thermal treatment at 700 °C due to the formation of interfacial oxide at the junction. (**b**) EQE spectra of *α*-FeSi(Al)/*n*-Si heterostructure solar cells annealed at 600 °C and 700 °C.

Impact of interface quality on the performance of silicide/silicon was also studied. The *α*-phase FeSi(Al)/*n*-Si solar cells were fabricated after thermal treatment of 22 nm thick FeSi(Al) layer at 600 °C and 700 °C. Figure 4(a) shows the current density-voltage characteristics of the solar cells annealed at 600 °C and 700 °C. The *V*_*oc*_ and *J*_*sc*_ of the solar cell decrease drastically from 425 mV and 18.5 mA/cm^2^ to 250 mV and 5.7 mA/cm^2^, respectively when annealing temperature is increased from 600 °C to 700 °C. Subsequently, the power conversion efficiency (PCE) of the device drops from 5.1% to 0.8%. This drastic drop in *V*_*oc*_ and *J*_*sc*_ could be due to the degradation in the material quality and the interface between Si and *α*-FeSi(Al) with the increase in the annealing temperature. Furthermore, to analyze the losses in the solar cell with the increase in the annealing temperature, EQE measurements were performed on the solar cells. Figure 4(b) shows the EQE spectra of *α*-FeSi(Al)/*n*-Si solar cells annealed at 600 °C and 700 °C. An EQE of over 45% is observed for *α*-FeSi(Al)/*n*-Si heterostructure solar cells annealed at 600 °C which drops to less than 10% when the annealing temperature is increased to 700 °C. The higher EQE response for the heterostructure samples annealed at 600 °C indicates a significantly increased charge separation and collection as compared to the samples annealed at 700 °C. The blue response for the samples annealed at 700 °C is zero indicating heavy recombination at the interface.

**Figure 5:**
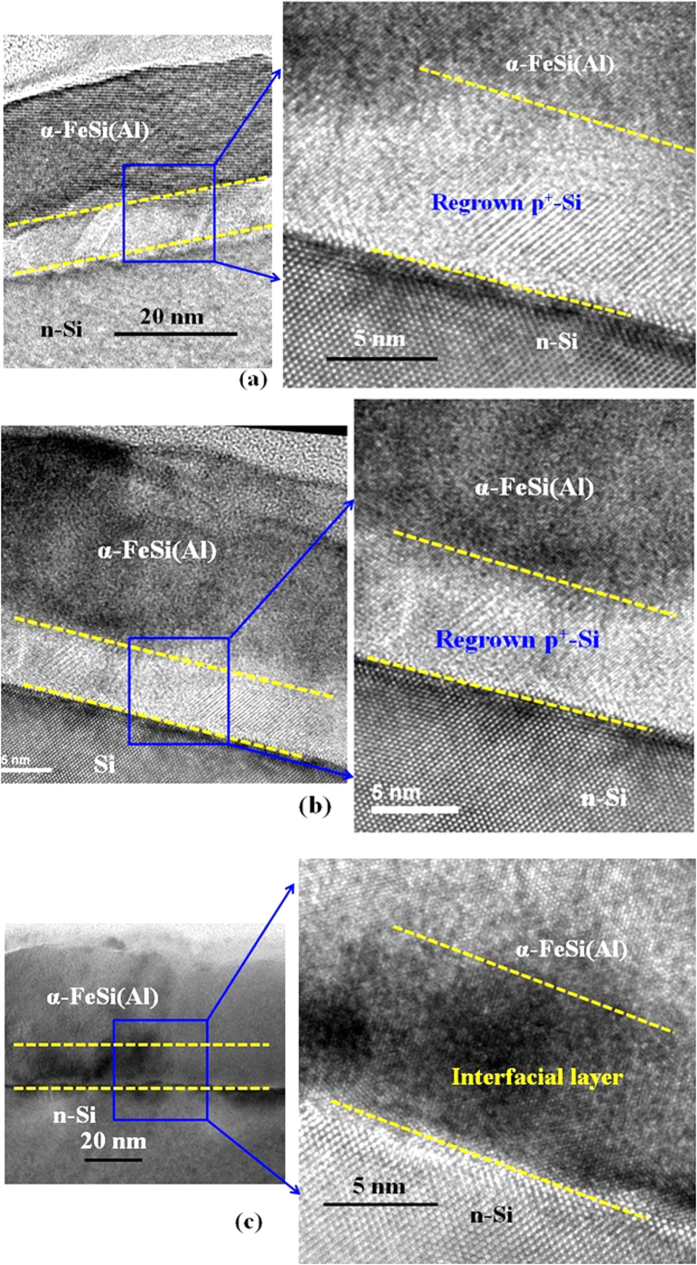
High-resolution transmission electron microscopy images of the *α*-phase FeSi(Al) ternary alloy on *n*-type silicon (100) substrate after thermal treatment of 600 °C for the thickness of (**a**) 22 nm and (**b**) 15 nm. Cross-sectional image shows formation of crystalline regrown Si after thermal treatment at 600 °C. (**c**) HRTEM images of 22 nm thick *α*-phase FeSi(Al) ternary alloy on *n*-type silicon (100) substrate after thermal treatment of 700 °C. Interface quality between *α*-phase FeSi(Al) layer and *n*-Si(100) substrate degraded after annealed at 700 °C.

It is worth to note that the performance of the *α*-FeSi(Al)/*n*-Si solar cells significantly depends on the thickness of *α*-FeSi(Al) layer and process temperature during the device fabrication. To understand the device performance and mechanism behind it, we have performed high-resolution transmission electron microscopy (HRTEM) analysis on the *α*-FeSi(Al)/*n*-Si structure. Figures 5(a,b) show HRTEM images of the α-phase Al alloyed FeSi_2_ of 22 nm and 15 nm grown on *n*-Si substrate after thermal treatment at 600 °C, respectively. A continuous uniform layer of *α*-FeSi(Al) film on the Si substrate is observed. Detailed observation of the HRTEM images (see Fig. 5(a,b)) reveals that there is a significant impact of the thickness of *α*-FeSi(Al) layer on the interface quality. A clear and sharp crystalline interface can be detected between the *α*-FeSi(Al) and the *n*-Si(100) substrate for the 22 nm thick *α*-FeSi(Al) layer (Fig. 5a). The *α*-phase FeSi(Al) is formed on the *n*-Si(100) substrate at 600 °C through the formation of thin regrown Si at the interface. Further detailed analysis of TEM images reveals that the thickness of regrown Si layer is ~10 nm. This regrown crystalline *p*^*+*^-Si (Al is *p*-type dopant) layer is formed during thermal treatment of the amorphous layer. During thermal annealing, thin film Al interlayer is mostly dissolved into amorphous layer to replace Si, which results in the formation of the heavy Al-doped crystalline-Si (*p*^*+*^-Si) interfacial layer by the expelled Si^22^,^34^. From the EDX analysis, it was found that the Al composition in the silicide layer and regrown silicon layer is ~10%. Thickness of regrown Si layer depends on the amorphous FeSi(Al) layer thickness. For the 15 nm thick FeSi(Al) layer, the regrown Si layer thickness reduces to ~5 nm and it is also not uniform (Fig. 5b).

Furthermore, the interface quality tends to degrade when the annealing temperature is increased to 700 °C (see Fig. 5c). According to the HRTEM analysis, it was found that the interface distorted with the presence of thin amorphous oxide layer of SiAl_x_Fe_y_O_z_^22^,^26^. The diffusion of Al into FeSi_2_ layer as well as into Si(100) substrate becomes significant when the annealing temperature is increased to 700 °C and beyond. This creates a deficiency of Al concentration at the interface which might lead to the distortion of interface with presence of thin amorphous layer of SiAl_x_Fe_y_O_z_^22^,^24^,^26^. In addition, *α*-FeSi(Al) tends to show polycrystalline nature at 700 °C which could also contribute to the reduction of the *V*_*oc*_ at high temperature^35^. The performance of the solar cell is mainly governed by the metal/silicon Schottky junction, as there was no formation of regrown silicon layer (Fig. 5c), where the cells were fabricated at 700 °C.

Formation of a highly Al-doped thin Si layer was also observed at *β*-FeSi_2_/*n*-Si(100) interface and *β*-FeSi_2_/*n*-ploy-Si interface^22^,^24^. However, *β*-FeSi_2_/*n*-Si(100), the regrown silicon layer is not uniform and also thickness of regrown silicon layer is thinner compared to the *α*-FeSi_2_ layer. This is mainly due to the difference of Al content in *α*-phase FeSi_2_ and *β*-phase FeSi_2_. The Al content in *α*-FeSi_2_ is 1.5 times higher compared with that of the *β*-FeSi_2_^34^. Since, higher Al content displaced larger number of Si atoms, as a result thicker regrown silicon layer was observed at the interface for α-phase FeSi_2_ compared with *β*-phase FeSi_2_. The solar cells performance governs through the formation of regrown highly doped crystalline layer of *p*^+^-Si on *n*-Si substrate for the device fabricated at 600 °C. Since, highly doped regrown silicon layer thickness reduced with the decrease of *α*-FeSi(Al) layer thickness, the open circuit voltage as well as performance of the device also reduced.

Even though the performance of solar cells governed by the formation of *p*^+^-*n*-Si homojunction for the device fabricated at 600 °C, it is essential to use *α*-FeSi_2_ layer to fabricate the device. To find the importance of *α*-FeSi_2_ layer, we have fabricated the Al/*n*-Si based devices without iron-silicide layer. Details of the device fabrication can be found in the supplementary content. The process temperature and surface cleaning was similar to the iron-silicide based devices (Fig. S1). We have found that the device performance is very poor. Open circuit voltage is ~60 mV and short-circuit current is less than 1 mA/cm^2^ (Fig. S2). Furthermore, we have also characterized the iron-silicde/silicon based device after removing iron silicide from the top surface (Fig. S3). The open-circuit voltage and short-circuit current are much lower compared with iron-silicide/silicon devices (Fig. S4). Thus, it is crucial to integrate *α*-phase FeSi_2_ layer with *n*-Si to fabricate Si-based photovoltaic devices using cost-effective and eco-friendly technique. However, it is also essential to optimize the *α*-FeSi(Al) layer and regrown Si layer thickness to achieve maximum efficiency of these solar cells.

It is also worth noting that the light falls on the *α*-FeSi(Al) surface of the solar cell. The *α*-FeSi(Al) is highly reflecting, resulting in the loss of current and efficiency. Therefore, use of effective anti-reflection coating could boost the performance of the solar cell further. On the other hand, the high refractive property of *α*-FeSi(Al) can be exploited, provided the structure of the solar cell is reversed to *n*-Si/*α*-FeSi(Al), where light will enter through Si. This makes *α*-FeSi(Al) highly promising candidate for poly-Si thin film and other band gap compatible thin film technologies which can use the internal reflection from α-FeSi(Al) surface. Even though, the efficiency of the reported device is lower compared with traditional single homojunction crystalline Si solar cells, it is worth to emphasize here that this is the first demonstrated result for such a device structure with efficiency more than 5% and it is comparable with other reported results for the similar device structure^11^,^13^,^14^,^15^,^16^,^17^,^37^,^38^. The photovoltaic parameters are compared with the reported results (Table 2). The average efficiency of the fabricated solar cells devices with 22 nm thick *α*-FeSi(Al) layer is 4.8% with standard deviation of 0.3. The novelty of this work lies in the fabrication and integration of *α*-FeSi(Al) with silicon using a simple and environmental-friendly approach at a low temperature using conventional sputter method. Furthermore, an optimum device structure with appropriate surface passivation is likely to enhance the performance of the device. Antireflective coatings (ARCs) have been used in solar cells to reduce the optical reflectance and enhance the power conversion efficiency of the cells^39^. An enhancement of 40% in power conversion efficiency was observed in the mono-crystalline Si solar cell with a triple-layer ARC^40^. Furthermore, a relative increase in power conversion efficiency of ~15.6% and ~22.8% was observed for single layer and double layer nanocrystals-embedded-ARC respectively, as compared to the solar cell with silicon-nitride based ARC^41^. It was also found that the metal nanoparticle induced surface resonance improved *p*-*n* junction based solar cells performance^42^,^43^. Recently, we have developed sputter grown *p*-type CuO which shows low reflection in the visible spectrum of solar radiation, it can be a suitable candidate for the surface passivation^44^. Thus, by employing the advance light trapping scheme along with proper surface passivation on *α*-FeSi(Al) layer and optimizing the regrown silicon layer thickness, it is possible to push the efficiency of the Al alloyed iron-silicide/silicon based solar cell well beyond ~12%.

## Conclusion

The interface quality of *α*-FeSi(Al)/*n*-Si was shown to depend on the annealing temperature and thickness of FeSi(Al) layer. A working solar cell device using *α*-FeSi(Al) and *n*-Si was demonstrated with promising photovoltaic properties. The solar cells exhibited an open circuit voltage, short-circuit current and a fill factor (FF) of 425 mV, 18.5 mA/cm^2^, and 63.5%, respectively, yielding a device efficiency of ~5.1%. Furthermore, photovoltaic parameters of *α*-phase FeSi(Al)/n-Si based solar cells were extracted using compact model. Interface quality and formation of thin film regrown silicon at the *α*-FeSi(Al)/silicon interface were found to be crucial for the performance of *α*-FeSi(Al)/*n*-type silicon based solar cells. Nanoscale surface engineering through the formation of regrown silicon layer can boost the performance of the device remarkably. There is a tremendous scope to improve the performance of the reported solar cell device with the introduction of effective surface passivation using anti-reflection coating on *α*-FeSi(Al) layer. This study shows new opportunities for the Si based photovoltaic technology using a sputter deposition method and low temperature processing for the development of a simple, sustainable, and los cost photovoltaic technology.

## Additional Information

**How to cite this article**: Kumar Dalapati, G. *et al.* Aluminium alloyed iron-silicide/silicon solar cells: A simple approach for low cost environmental-friendly photovoltaic technology. *Sci. Rep.* **5**, 17810; doi: 10.1038/srep17810 (2015).

## Supplementary Material

Supplementary Information

## Figures and Tables

**Table 1:** 

Thickness of α-FeSi(Al) (nm)	35.0	22.0	15.0
*J*_*sc*_ (mA/cm^2^)	13.2	18.5	22.9
*V*_*oc*_ (mV)	406.0	425.0	348.2
*FF* (%)	64.4	63.5	53.8
*PCE* (%)	3.5	5.1	4.3
*n*	1.8	1.8	2.2
*R*_*s*_ (Ωcm^2^)	1.1	1.2	1.3
*R*_*sh*_ (kΩcm^2^)	2.9	3.3	3.6

Photovoltaic parameters such as short-circuit current (*J*_*sc*_), open-circuit voltage (*V*_*oc*_), fill-factor (*FF*), power conversion efficiency (*PCE*), ideality factor (*n*), series resistance(*R*_*s*_), and shunt resistance (*R*_*sh*_) are presented for the solar cells with different thickness of *α*-FeSi(Al) layer on *n*-Si substrates.

**Table 2:** Comparison of photovoltaic performance of *α*-FeSi(Al)/*n*-Si solar cells with the reported results.

Device Structure	*J*_*sc*_ (mA/cm^2^)	*V*_*oc*_ (mV)	*FF* (%)	*Eff* (%)	Refs.
*p*-CuO/*n*-Si	6.4	494	32	1.0	[11]
PEDOT:PSS/*n*-SiNW	28.5	524	63.5	9.5	[12]
Graphene/*n*-Si	0.15	420	46	1.5	[13]
Graphene/*n*-Si	23.8	460	40	4.4	[14]
Graphene/*n*-SiNW	11.2	503	50.6	2.8	[15]
CNT/*n*-Si	26	524	53	7.4	[16]
MoS_2_/*p*-Si	22.4	410	57.3	5.2	[17]
*n*-SiNW/*p*-Si	24.4	460	63.0	7.0	[38]
*p*-CuO/*n*-Si	6.1	498	39.9	1.2	[37]
α-FeSi(Al)/*n*-Si	18.5	425	63.5	5.1	This work
