# Supplementary material for: Aluminium alloyed iron-silicide/silicon solar cells: A simple approach for low cost environmental-friendly photovoltaic technology
Source: Sci Rep. 2015 Dec 3;5:17810. doi: 10.1038/srep17810 (PMC4668570; doi:10.1038/srep17810)
Supplement: Supplementary Information [file srep17810-s1.doc]

***Aluminium alloyed iron-silicide/silicon solar cells: A simple approach for low cost environmental-friendly photovoltaic technology***

Goutam Kumar Dalapati*, Saeid Masudy-Panah, Avishek Kumar, Cheng Cheh Tan, Hui Ru Tan, and Dongzhi Chi

Institute of Materials Research and Engineering, A*STAR (Agency for Science, Technology and Research), 3 Research Link, Singapore 117602

*corresponding author, E-mail: [dalapatig@imre.a-star.edu.sg](mailto:dalapatig@imre.a-star.edu.sg)

To understand the impact of *α*-phase Al alloyed iron-silicide, we have fabricated solar cells without iron-silicide. Thin film of Al was deposited on the *n*-Si surface and annealed at 600^o^C-700^o^C for 1 min in nitrogen ambient. Figure S1 shows the schematic diagram of Al/*n*-Si solar cells. Current-voltage characteristics of the solar cells were presented in the figure S2. The open-circuit voltage and short-circuit current significantly lower compared with the device fabricated using iron-silicide.


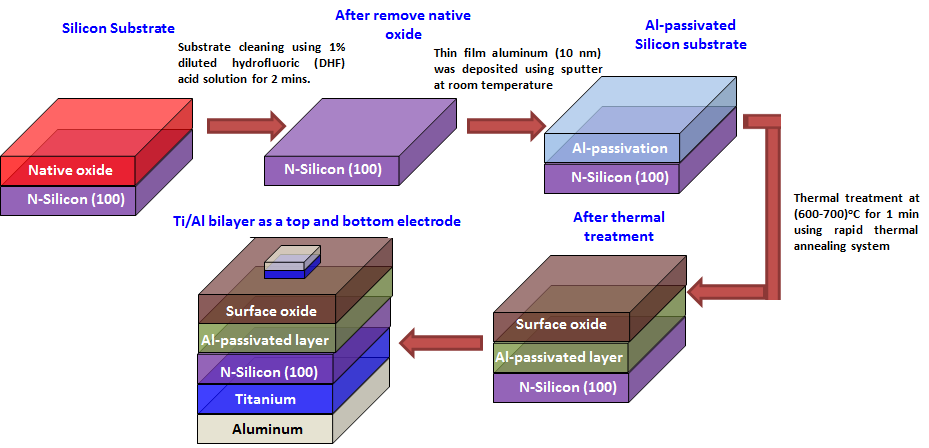


Figure S1: Schematic diagram of Al/*n*-Si structure with top and bottom electrode.

Figure S2: Current-voltage characteristics of Al/*n*-Si structure after thermal treatment at 600^o^C, 650^o^C, and 700^o^C for 1 min in nitrogen ambient.

Furthermore, we have also fabricated the *α*-phase Al alloyed iron-silicide based solar cells using same process reported in the paper. After thermal treatment of the silicide layer, we have removed the iron silicide layer completely by using HF solution. Figure S3 shows the schematic diagram of the device. Current-voltage characteristics were presented in the figure S4. It is worth to note that the performance is very poor for both cases compared to the silicide based device. Thus, it is necessary to integrate *α* -phase iron-silicide with *n*-type Si.


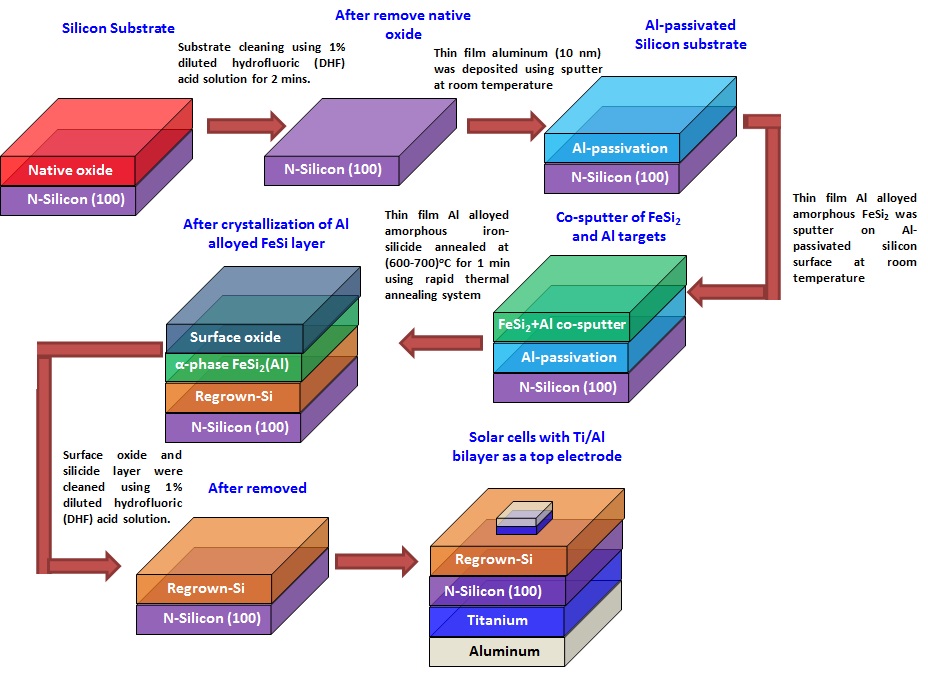


Figure S3: Schematic diagram of regrown *p*^+^-Si/*n*-Si structure with top and bottom electrode.

Figure S4: Current-voltage characteristics of Regrown *p*^+^-Si/*n*-Si solar cells.
